# CBX2 identified as driver of anoikis escape and dissemination in high grade serous ovarian cancer

**DOI:** 10.1038/s41389-018-0103-1

**Published:** 2018-11-26

**Authors:** Lindsay J. Wheeler, Zachary L. Watson, Lubna Qamar, Tomomi M. Yamamoto, Miriam D. Post, Amber A. Berning, Monique A. Spillman, Kian Behbakht, Benjamin G. Bitler

**Affiliations:** 1 Division of Gynecologic Oncology, Department of Obstetrics and Gynecology, University of Colorado Anschutz Medical Campus, Aurora, CO USA; 2 Division of Reproductive Sciences, Department of Obstetrics and Gynecology, University of Colorado Anschutz Medical Campus, Aurora, CO USA; 3 Department of Pathology, University of Colorado Anschutz Medical Campus, Aurora, CO USA; 4 Gynecologic Oncology, Texas A&M University Medical School, Baylor University Medical Center, Dallas, TX USA

## Abstract

High grade serous ovarian carcinoma (HGSOC) is often diagnosed at an advanced stage. Chromobox 2 (CBX2), a polycomb repressor complex subunit, plays an oncogenic role in other cancers, but little is known about its role in HGSOC. We hypothesize that CBX2 upregulation promotes HGSOC via induction of a stem-like transcriptional profile and inhibition of anoikis. Examination of Gene Expression Omnibus (GEO) datasets and The Cancer Genome Atlas (TCGA) established that increased *CBX2* expression conveyed chemoresistance and worse disease-free and overall survival. In primary HGSOC tumors, we observed CBX2 expression was significantly elevated compared to benign counterparts. In HGSOC cell lines, forced suspension promoted CBX2 expression. Subsequently, CBX2 knockdown inhibited anchorage-independent proliferation and potentiated anoikis-dependent apoptosis. Furthermore, CBX2 knockdown re-sensitized cells to platinum-based chemotherapy. Forced suspension promoted increased ALDH activity and *ALDH3A1* expression and CBX2 knockdown led to a decrease in both ALDH activity and *ALDH3A1* expression. Investigation of CBX2 expression on a HGSOC tissue microarray revealed CBX2 expression was apparent in both primary and metastatic tissues. CBX2 is an important regulator of stem-ness, anoikis escape, HGSOC dissemination, and chemoresistance and potentially serves as a novel therapeutic target.

## Introduction

Epithelial ovarian cancer is the deadliest gynecologic malignancy and annually accounts for over 220,000 deaths worldwide^1^. In the US, over 22,000 new cases of ovarian cancer are diagnosed each year and over 14,000 women succumb to the disease^2^. The majority of these cases are classified as high-grade serous ovarian carcinoma (HGSOC). HGSOC tends to be diagnosed at a late stage, when cancer has already spread beyond the pelvis, and will recur in the majority of cases^1^. Current evidence suggests that HGSOC originates from transformed secretory fallopian tube epithelium (FTE) cells located on the fimbriated end of the fallopian tube^3–5^. Precursor lesions, defined by *TP53* mutations, include serous tubal intraepithelial carcinoma (STIC), which is focal and displays a cytologic appearance similar to HGSOC^3–5^. Cells within STIC lesions demonstrate anoikis resistance or anchorage-independent cell survival by exfoliation from the fallopian tube-associated extracellular matrix and dissemination to the ovary and/or peritoneum. Ovarian, fallopian, and primary peritoneal carcinomas differ from other epithelial cancers that metastasize to distant sites predominantly via the circulatory or lymphatic systems (e.g., breast, endometrial) by spreading directly to the ovaries and the abdominal cavity independent of the lymphatic or vascular system. As HGSOC cells spread to the abdominal cavity they promote the production of ascites, a collection of intra-peritoneal fluid containing immune cells, tumor cells, and cytokines, along with other cellular and acellular factors^6^. Notably, the prevalence of ascites is directly correlated to disease stage. For instance, 89% of stage III/IV patients present with some degree of ascites^7^. Tumor cells within ascites are hypothesized to be a subpopulation of cells that contribute to disseminated, recurrent, and chemoresistant disease^6^. However, the genetic drivers of HGSOC dissemination and anchorage-independent survival remain unclear.

A significant proportion of “stem”-like cells have been detected in the ascites fluid associated with HGSOC^8,9^. One group of transcriptional repressors, the polycomb group (PcG) of proteins, are candidates for producing and maintaining this “stemness” as they have been shown to inhibit cellular differentiation and maintain a stem-like transcriptional program. PcG proteins assemble in two main Polycomb repressive complexes, PRC1 and PRC2 [reviewed in ref. ^10^]. PRC1 and 2 epigenetically repress pro-differentiation and tumor suppressor genes, and are important in several cancer types including prostate, breast, and HGSOC^10,11^. Epigenetic “readers”, known as chromobox (CBX) proteins, play a critical role in PRC1 repressive activity by recognizing methylated histones through their chromobox domain. In 2014, Clermont et al. initially identified an oncogenic role for CBX2 through a genotranscriptomic meta-analysis in human cancers. In breast and prostate cancers, they reported that CBX2 upregulation and amplification significantly correlated with metastatic progression and lower overall survival^12–14^. CBX2 depletion reduced cell viability and promoted apoptosis in metastatic prostate cancer, suggesting that CBX2 drives key regulators of cell proliferation and metastasis^15^. Gui et al. evaluated the role of 12 PcG proteins in primary and recurrent ovarian cancer and found that immunohistochemistry (IHC) demonstrated significantly higher levels of CBX2 expression in recurrent tumors compared to primary tumors at presentation (primary ovarian tissue at presentation *n* = 100, recurrent disease at relapse *n* = 50, *p* < 0.001)^16^. However, the role of CBX2 in HGSOC progression is unknown.

In this study, we demonstrate that CBX2 is overexpressed in primary HGSOC tumors and that CBX2 protein is upregulated in HGSOC cells grown in an anchorage-independent fashion (forced suspension). In a primary human HGSOC tumor microarray we observed high CBX2 expression in a majority of specimens. We show that the loss of CBX2 inhibits proliferation, reduces stemness, and increases cisplatin sensitivity. These studies suggest that CBX2 could be an important therapeutic target in HGSOC.

## Results

### CBX2 is upregulated in high grade serous ovarian cancer and is associated with poor survival

**Fig. 1:**
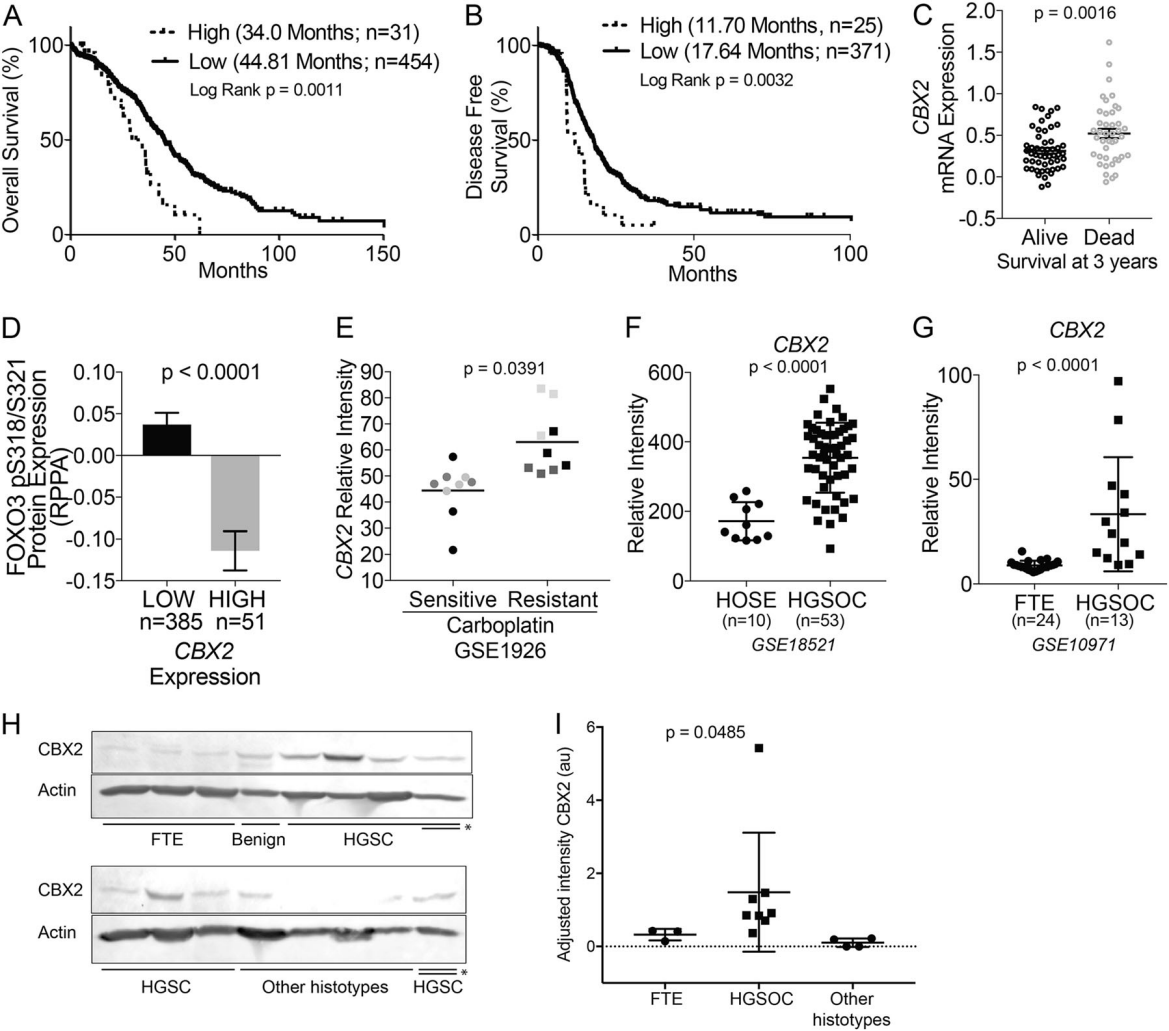
CBX2 is overexpressed in high grade serous carcinoma and portends poor prognosis. **a** Overall survival analysis comparing expression of CBX2 (High = mRNA expression > 1.5 standard deviation; Low = SD < 1.5(=)). High (*n* = 31) vs. Low (*n* = 454) CBX2 expression (mean survival 34.0 months vs. 44.81 months, Log Rank *p* = 0.0011). **b** Same as (**a**), disease-free survival analysis, again comparing upregulation of CBX2, defined as above (High *n* = 25, Low *n* = 371). Increased expression of CBX2 was associated with statistically significant decreased disease-free survival (11.70 months vs. 17.64 months, Log Rank *p* = 0.0032). Number of patients differ from (**a**) due to available data within TCGA. **c** Examination of CBX2 mRNA expression in HGSOC patients alive (*n* = 51) and dead (*n* = 42) at 3 years after diagnosis. Data obtained from HGSOC Tothill Cohort^47^. Statistical test = two-sided *t*-test, *F* test *p* = 0.0057 **d** Correlation of *CBX2* expression with FOXO3_PS318/321 in TCGA (“Provisional data” with RPPA data, *n* = 435) tumors (High = upper quartile and Low = bottom three quartiles). Statistical test = two-sided *t*-test, *F* test *p* < 0.0001. **e** Bioinformatic analysis evaluating relative intensity of *CBX2* in high grade serous ovarian carcinoma (HGSOC) cases described as platinum sensitive (*n* = 3) or resistant (*n* = 3). Each tumor (color-coded) was examined in triplicate. Statistical test examined average CBX2 intensity for each tumor (GSE1926; one-sided *t*-test *p* = 0.0391, *F* test *p* = 0.29). **f** Relative intensity of CBX2 in benign human ovarian surface epithelium (HOSE, *n* = 10) compared to HGSOC, *n* = 53 (GSE18521; *t*-test *p* < 0.0001, *F* test *p* < 0.0001). **g** Relative intensity of CBX2 in fallopian tube epithelium (FTE, *n* = 24) compared to HGSOC, *n* = 13 (GSE10971; two-sided *t*-test *p* < 0.0001, *F* test *p* < 0.0001). **h** Protein lysates generated from the primary tissue of FTE, HGSOC, and mixed histotypes. Protein utilized for immunoblot against CBX2. (beta-actin = loading control) **i** Densitometric analysis of immunoblots. Intensities were normalized between immunoblot by indicated (*) sample (FTE vs. HGSOC, one-sided Rank Sum *p* = 0.0333)

We examined *CBX2* expression in HGSOC in several publicly available datasets (Gene Expression Omnibus; GEO Dataset and The Cancer Genome Atlas; TCGA). High expression of *CBX2* in TCGA HGSOC samples conveyed both a significantly worse disease-free survival (DFS; 11.7 vs. 17.6 months, Log-rank test *p*-value 0.00316) and overall survival (OS; 34 vs. 44.8 months, Log-rank test *p*-value 0.00116) (Fig. 1a, b)^17^. In an independent HGSOC data set, high CBX2 expression was associated with poorer survival at 3 years (Fig. 1c). Further correlation of *CBX2* expression with protein expression via reverse-phase protein array (RPPA) found several proteins significantly enriched or depleted in *CBX2* high expressing tumors (Supplementary Table 1). Notably, phosphorylated serine 318 and 321 FOXO3, a known tumor suppressor^18,19^, was depleted in tumors with high CBX2 expression (Fig. 1d). Additionally, using the GEO Dataset (GSE1926), a comparison of platinum sensitive HGSOC tumors to platinum resistant HGSOC tumors, demonstrated an increase in *CBX2* in resistant tumors, further supporting the association between CBX2 and more aggressive HGSOC (Fig. 1e).

Ovarian surface and FTE are proposed to be the precursor cells for HGSOC; more recent data strongly support FTE as the predominant site of origin^3–5^. Comparing *CBX2* expression in ovarian surface epithelium or FTE to *CBX2* expression in HGSOC, we observed that *CBX2* was significantly higher in HGSOC (Fig. 1f, g) (GSE18521 and GSE10971). To confirm the extent of these findings we examined protein derived from primary tissues of four FTE and benign tissues and seven HGSOC tumors collected through the University of Colorado Gynecologic Tumor and Fluid Bank (GTFB) (Fig. 1h; Supplementary Table 2). Utilizing densitometry, CBX2 expression was observed to be significantly higher in HGSOC primary tumor compared to FTE or benign tissues (Fig. 1i, Rank-sum test *p* value 0.0333). In other ovarian cancer histosubtypes, we noted a lack of CBX2 expression suggesting the oncogenic effects of CBX2 are HGSOC specific. Taken together, these data demonstrate that CBX2 upregulation in HGSOC is associated with poorer prognosis, repression of the FOXO3 tumor suppressor, and is possibly linked to chemoresistance.

### CBX2 is upregulated in tumor cells in suspension

**Fig. 2:**
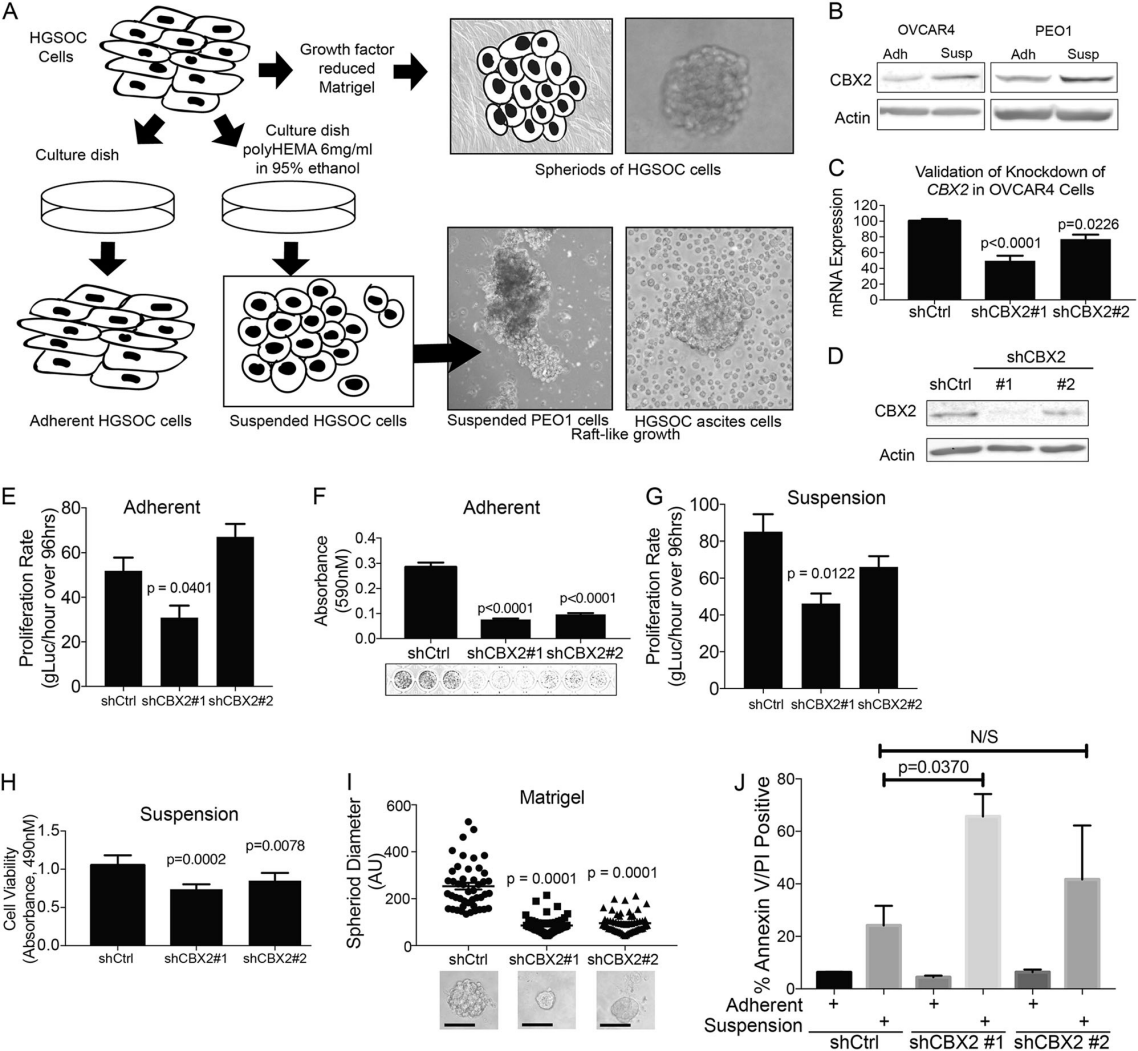
Inhibition of CBX2 impairs HGSOC cell proliferation. **a** Model describing basic protocol for establishing adherent, suspension, and spheroid growth environments. For adherent and suspension, two verified high grade serous ovarian carcinoma cell lines were initially grown on tissue culture plastic, then distributed to normal tissue culture dishes (adherent) and polyHEMA coated culture dish (suspension) growth environments. Distributed at 1:3 or 1:5 ratio to account for forced suspension induced cell death. Photographs show PEO1 cells after 7 days in suspension (left) and HGSOC cells directly derived from patient ascites. For spheroid formation, cells were grown in 3D in Matrigel for 12 days. A representative image of a resulting spheroid is shown at upper right. **b** Immunoblots against CBX2 protein from OVCAR4, and PEO1 cells grown in adherent and suspension settings (described in (**a**)) over 7 days. **c** RT-qPCR for *CBX2* in OVCAR4 cells transduced with small hairpin RNA (shRNA) specific for *CBX2*. Representative mRNA expression of *CBX2* in shControl, shCBX2#1, and shCBX2#2. Statistical test = ANOVA. **d** Immunoblots against CBX2 protein derived from OVCAR4 shControl, shCBX2#1, and shCBX2#2 transduced cells (beta-actin = loading control). **e** Proliferation assay of OVCAR4 cells with CBX2 knockdown and shControl, grown in adherent setting (tissue culture plastic) over 96 h, evaluated using gLuc activity. Statistical test = ANOVA. **f** Same as (**e**), crystal violet staining and subsequent measurement of absorbance at 590 nm. Images of representative of stained cells from shControl, shCBX2#1, and shCBX2#2. Statistical test = ANOVA. **g** Proliferation assay of OVCAR4 cells with CBX2 knockdown and shControl, grown in suspension setting (poly-HEMA coated tissue culture plastic) over 96 h, evaluated using gLuc activity. Statistical test = ANOVA. **h** Same as (**g**), but cell viability was assessed via MTT after 96 h. Statistical test = ANOVA. **i** OVCAR4 cell lines (shControl, shCBX2 #1 and #2) grown in 3D using Matrigel over 12 days, leading to spheroid growth. Representative images of transduced OVCAR4 cells below. Scale bar = 100 μm. Spheroids measured across horizontal diameter. Diameter mean calculated from measurements of 50 spheroids per cell type. Statistical test = ANOVA. **j** OVCAR4 cells with CBX2 knockdown and shControl grown in adherent or suspension. Cells were subjected to AnnexinV/PI apoptosis assay. Statistical test = two-sided *t*-test, *F* test *p* = 0.9291. Error bars = S.E.M.

HGSOC is unique compared to other solid types in its tendency to directly seed and disseminate throughout the peritoneal cavity, which requires an escape from anoikis, an anchorage-independent cell death. To determine whether CBX2 plays a role in HGSOC tumor cell’s ability to survive without anchorage, or in a suspended setting, we examined the role of CBX2 on HGSOC growth in suspension. A forced suspension setting was achieved by plating cells on polyHEMA-coated tissue culture dishes (Fig. 2a)^20^.

The high grade serous ovarian cancer cell lines OVCAR4, PEO1, and OVCAR8 were grown in adherent and suspended settings (Fig. 2a). During the course of optimizing our model, we noted that OVCAR4 and PEO1 cells grown in suspension demonstrated a morphology and organization similar to cells derived from primary ascites fluid (data not shown and Fig. 2a). After culturing HGSOC cell lines for 7 days in a forced suspension, condition protein was extracted and subsequently used for immunoblots against CBX2. For all cell lines examined, CBX2 expression was increased in cells grown in suspension conditions (Fig. 2b; Supplementary Figure 1A). This observation serves as the foundation for our work, as the phenotype we describe is reinforced by both cell survival in suspension, as well as intact expression of CBX2.

### CBX2 knockdown inhibits proliferation

To further elucidate the role of CBX2 in HGSOC, we evaluated the impact of CBX2 modulation in PEO1, OVCAR4, and OVCAR8 HGSOC cell lines. One of the two independent small hairpin RNAs (shRNA) specific for CBX2 or a control (shControl) were transduced into OVCAR4, PEO1, and OVCAR8 cells. CBX2 knockdown was confirmed via quantitative PCR (qPCR) and immunoblots, with approximately 60% knockdown in the presence of shCBX2#1 and 30% knockdown in the presence of shCBX2#2 (Fig. 2c, d; Supplementary Figure 1B-D). PEO1 and OVCAR4 CBX2 knockdown cells were subjected to proliferation assays in 2D tissue culture dishes and in suspension as demonstrated in Fig. 2a. To assess changes in proliferation, cells were transduced with a retrovirus specific for Gaussia luciferase (gLuc)^21^. Changes in gLuc activity were shown to be directly correlated with cell number (Supplementary Figure 1E). OVCAR4 and PEO1 CBX2 knockdown cells were plated in adherent (2D) conditions and for 96 h gLuc activity was measured every 24 h. As a confirmatory assay, colony formation was examined in parallel on cells grown in 2D. CBX2 knockdown cells had a significantly reduced rate of gLuc activity and reduced colony formation (Fig. 2e, f and Supplementary Figure 1F). OVCAR4 and PEO1 CBX2 knockdown cells were plated in forced suspension conditions and gLuc activity was monitored every 24 h for 96 h and cell viability was determined for cells grown in forced suspension. Similar to adherent conditions, CBX2 knockdown had a significantly reduced rate of gLuc activity and viability (Fig. 2g, h and Supplementary Figure 1G). HGSOC grown on extracellular matrix more closely recapitulates the tumor microenvironment^22^, therefore OVCAR4 and PEO1 shControl and shCBX2 (#1 and #2) cells were grown in matrigel for 12 days. Spheroid diameter was measured for at least 50 spheroids in each condition and used as a surrogate for cell number^23^. CBX2 knockdown significantly reduced spheroid size compared to shControl control cells (Fig. 2i and Supplementary Figure 1H). In all culture conditions examined, we observed that CBX2 knockdown significantly decreased HGSOC cell viability. Upon closer examination of forced suspension conditions, we observed in OVCAR4, PEO1, and OVCAR8 cells that CBX2 knockdown potentiated anoikis, anchorage-independent cell death (Fig. 2j and Supplementary Figure 1I-J). These data indicate that CBX2 is important in promoting HGSOC cell proliferation and protecting against anoikis.

### Tissue microarray supports a role for CBX2 in tumor progression

**Fig. 3:**
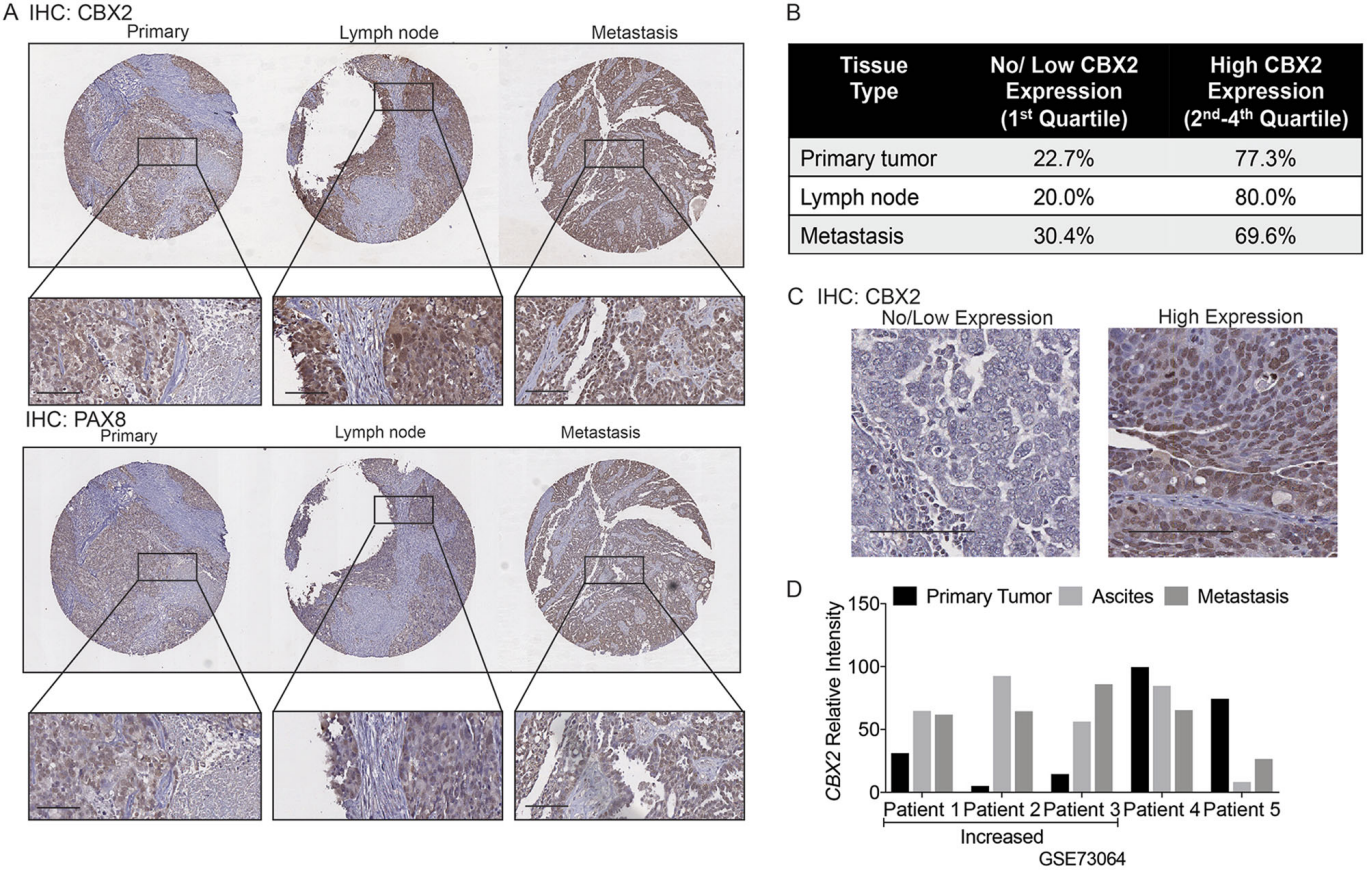
CBX2 expressed in advanced HGSOC. **a** Immunohistochemistry (IHC) against CBX2 and PAX8 utilizing a HGSOC tissue microarray (TMA) of 24 matched patient samples. Representative images of matched patient samples shown. Initial images shown at 3×, inset of images at 18×. Scale bar = 100 μm. **b** TMA analyzed using PAX8 staining a control for tumor area followed by analysis with Image Scope software to determine relative CBX2 tumor-associated intensity. Level of expression divided into quartiles. Breakdown of samples into 1st quartile (low or no expression) compared to 2nd–4th quartiles (moderate or high expression). Analysis by two board-certified pathologists (authors: M.D.P. and A.A.B.) confirmed Aperio findings. Values displayed in table, CBX2 expression seems to be consistent between tissue types. **c** Representative images of “High” and “No/Low” CBX2 expression. Scale bar = 100 μm. **d** Bioinformatic analysis evaluating relative intensity of *CBX2* in high grade serous ovarian carcinoma (HGSOC) cases with matched primary tumor (black bars), ascites-associated tumor cells (Ascites, light gray bars), and distant metastasis (dark gray bars) (*n* = 5, GSE73064)

In order to correlate the CBX2 in vitro findings to clinically relevant specimens, we utilized a tissue microarray (TMA) of HGSOC that recapitulated tumor progression. The TMA contained 24 primary tumors, with matched lymph node or distant metastases (Supplementary Table 3). IHC was optimized and performed using a previously published CBX2 antibody, predicted to preferentially stain the nucleus (Fig. 3a and Supplementary Figure 2)^13^. In parallel the TMA was stained with PAX8, a marker for Müllerian origin (Fig. 3a). CBX2 and PAX8 stained TMAs were scanned using the Aperio system and annotated based on the PAX8 staining profile. Objective software-based approaches were utilized to score and analyze the scanned and annotated CBX2 TMAs. The level of expression was compared between primary tumors, metastases, and lymph nodes and quartiles were calculated. Across all three tissue types, 20–30% of the specimens were considered “no or low expression” (first quartile) compared to 69–80% of the tissues that were moderate to high expression (second through fourth quartile) (Fig. 3b, c). Results from Aperio analysis were confirmed via manual scoring by two independent board-certified pathologists. Similarly, when the intensity of staining was evaluated between matched samples there was no significant change between matched samples. Given that the tissues were derived from HGSOC patients with advanced disease, as demonstrated by both metastatic and lymph node involvement, this would suggest that CBX2 expression potentially correlates with a more aggressive disease. We next examined *CBX2* expression in matched primary tumor, ascites-associated tumor cells, and metastatic tumors from five HGSOC patients (GSE73064). In two of the five patients, CBX2 expression was high in the primary tumor, however three of the five patients had an increase of *CBX2* in metastatic and ascites samples compared to primary tumor (Fig. 3d). Consistently, these findings align with our bioinformatics analysis indicating that increased CBX2 expression is associated with decreased survival and more advanced disease.

### CBX2 expression is associated with chemoresistance

**Fig. 4:**
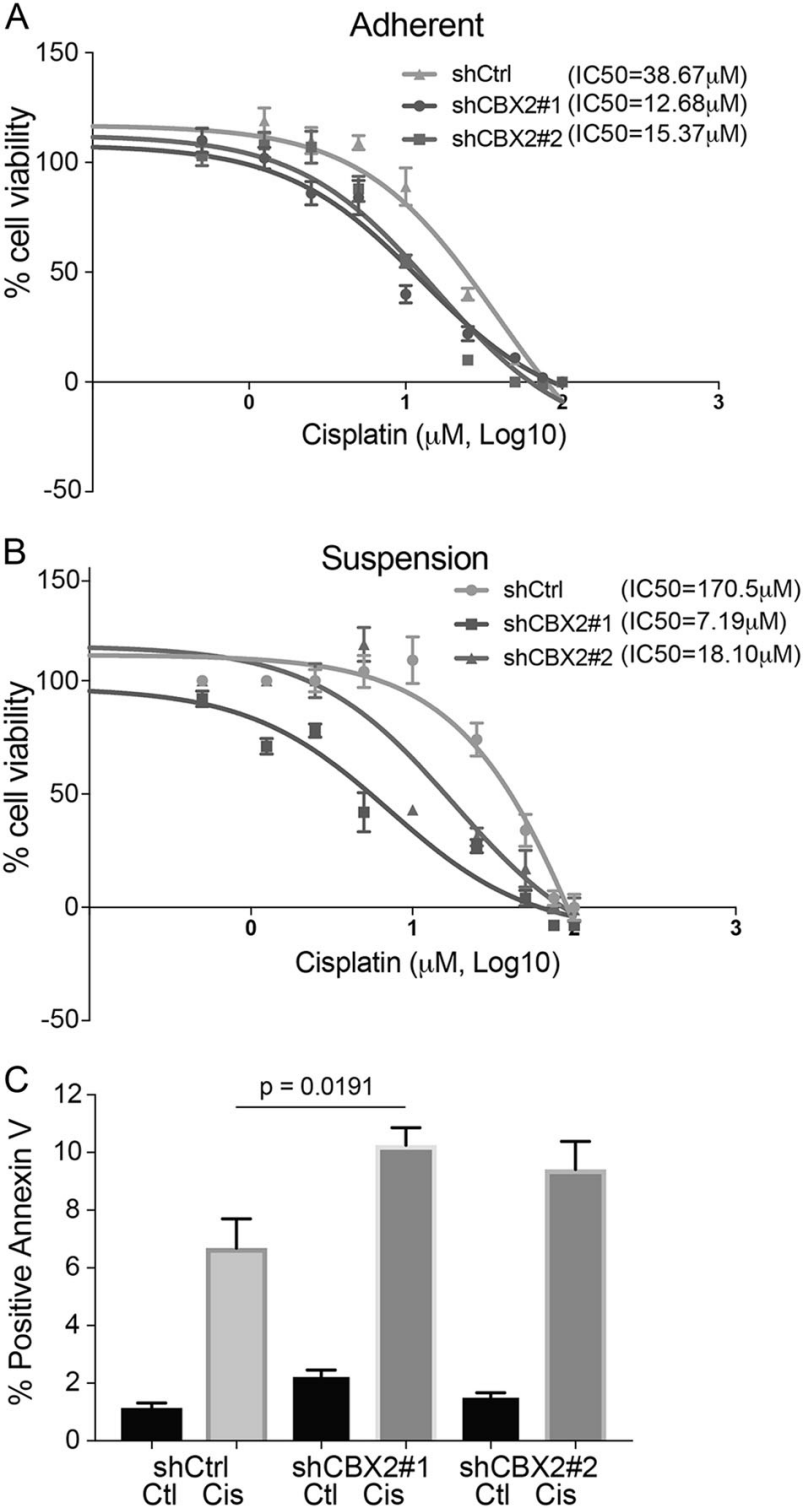
Loss of CBX2 sensitizes HGSOC to chemotherapy. **a** OVCAR4 shControl, shCBX2#1, and shCBX2#2 in 96-well plates treated over 24 h with increasing dose of cisplatin (0.5–100 µM). Percent cell viability was measured using the MTT assay and the half maximal inhibitory concentration (IC_50_) calculated. **b** Similarly to (**a**), OVCAR4 knockdown cell lines were grown in low adherent 96-well plates (forced suspension) and treated with increasing doses of cisplatin over 24 h and percent cell viability measured with MTT for calculation of IC_50_. **c** Annexin/V apoptosis assay of OVCAR4 cells grown in adherent setting with shControl, shCBX2#1 or #2 treated with cisplatin (10 µM) compared to untreated control. Percent Annexin positive cells are shown. Statistical test = ANOVA. Error bars = S.E.M.

A majority of HGSOC patients treated with platinum-based (i.e., carboplatin) chemotherapy develop chemoresistance. A comparison of carboplatin sensitive HGSOC tumors to platinum resistant tumors demonstrates an increase in *CBX2* in platinum resistant tumors (GSE1926) (Fig. 1c). Based on these data and our finding that CBX2 protects against anoikis, we hypothesized that CBX2 attenuates chemotherapy response. To test this hypothesis, CBX2 knockdown OVCAR4, PEO1, and OVCAR8 cells were grown in an adherent setting for 24 h and subsequently treated with increasing doses of cisplatin. Assessment of cell viability showed that in OVCAR4, PEO1, and OVCAR8, shCBX2 cell lines were significantly more chemosensitive than the shControl cells (Fig. 4a and Supplementary Figure 3A-B). For example, CBX2 knockdown OVCAR4 cells had an IC_50_ of 12.68 μM in shCBX2#1 and 15.37 μM in shCBX2#2 compared to 38.67 μM for the control with intact CBX2 (Fig. 4a). Haley et al.^24^ reported the OVCAR4 cisplatin IC_50_ to be approximately 6 μM, however unlike this report we did not allow cells to recover 72 h following cisplatin treatment which likely accounts for this discrepancy. CBX2 knockdown OVCAR4, PEO1, and OVCAR8 cells were grown in suspension and dosed with cisplatin. OVCAR4 shCBX2 cell lines grown in suspension were found to be re-sensitized to platinum treatment with an IC_50_ of 7.19 μM (shCBX2#1) and 18.10 μM (shCBX2#2) compared to the control with intact CBX2 at an IC_50_ of 170.50 μM (Fig. 4b). Although not as robust as OVCAR4 cells, CBX2 knockdown also sensitized OVCAR8 and PEO1 cells to cisplatin (Supplementary Figure 3C-D). Notably, in OVCAR4 cells we observed a 4.47-fold increase in the cisplatin IC_50_ in suspension cells compared to the adherent cells. In addition, we further confirmed that in OVCAR4, loss of CBX2 lead to increased cisplatin-induced apoptosis measured with Annexin V/PI (Fig. 4c). These findings strongly support the hypothesis that anoikis-resistance, demonstrated by survival in suspension, and intact CBX2 expression, both promote chemoresistance.

### CBX2 regulation of autophagy, apoptosis, and EMT-related genes

**Fig. 5:**
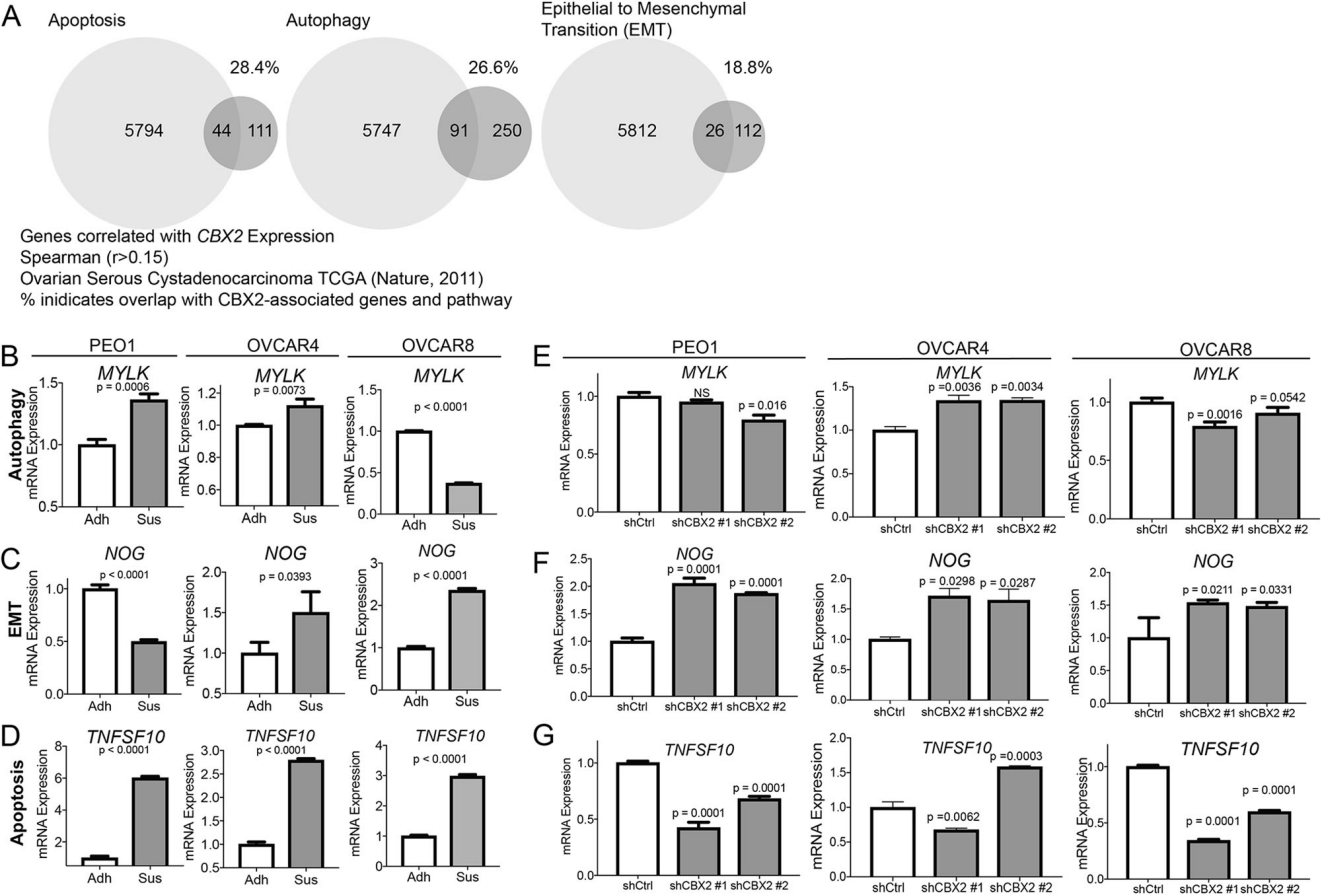
Correlation of CBX2 expression and autophagy, apoptosis, and EMT. Utilizing the TCGA (HGSOC, Nature, 2011) dataset, *CBX2* expression was correlated to mRNA expression of all genes and 5838 genes (CBX2-associated genes) were identified to have a Spearman correlation of greater than *r* = 0.15. **a** The 5838 genes were cross-referenced with published gene sets for “Apoptosis”, “Autophagy”, and “Epithelial to Mesenchymal Transition (EMT).” Percentage indicates overlap with gene lists. PEO1, OVCAR4, and OVCAR8 cells were grown in adherent (Adh) or suspension (Sus) for 7 days, RNA was extracted, and used for RT-qPCR against MYLK (**b**), NOG (**c**), and TNFSF10 (**d**). Statistical test = two-sided *t*-test. shControl and shCBX2 #1 and 2 PEO1, OVCAR4, and OVCAR8 cells were grown in suspension, RNA was extracted, and used for RT-qPCR against MYLK (**e**), NOG (**f**), and TNFSF10 (**g**). Statistical test = ANOVA. Error bars = S.E.M.

Chemoresistance and dissemination of ovarian cancer cells are associated with changes in apoptosis, epithelial to mesenchymal transition (EMT), and autophagy^25–28^ [reviewed in ref. ^29^]. The PcG is an epigenetic complex that is responsible for transcriptional reprogramming through histone modification thus CBX2 could regulate a variety of genes. Utilizing HGSOC TCGA data we generated a list of potential CBX2 target genes through examination of mRNA correlations (Spearman *r* > 0.15, 5838 genes, Supplementary Table 4). Utilizing published gene sets for EMT, autophagy, stemness, and apoptosis we cross-referenced the CBX2-associated genes (Supplementary Table 4)^30,31^. CBX2-associated genes accounted for 18.8–28.4% of genes in the respective pathways (Fig. 5a). We selected a gene from each pathway and in OVCAR4, OVCAR8, and PEO1 observed that the genes were differentially regulated in adherent vs. suspension culture conditions. For instance, in two of the three cell lines an autophagy-related gene, *MYLK*^32^, and EMT-related gene, *NOG*^33^, were significantly differentially regulated in suspension (Fig. 5b, c). Furthermore, we examined an apoptosis-related gene, *TNFSF10*, and observed that it was upregulated in all three HGSOC cells lines when grown in suspension (Fig. 5d). Subsequently, CBX2 knockdown cells grown in suspension differentially regulate *MYLK*, *NOG*, and *TNFSF10* (Fig. 5e–g). The differential expression observed correlated with the level of CBX2 knockdown (Fig. 2c and Supplementary Figure 1B-D). These findings suggest that in the context of anoikis CBX2 regulates several pathways, including autophagy, apoptosis, and EMT.

### Forced growth in suspension and increased CBX2 leads to a stem-like phenotype

**Fig. 6:**
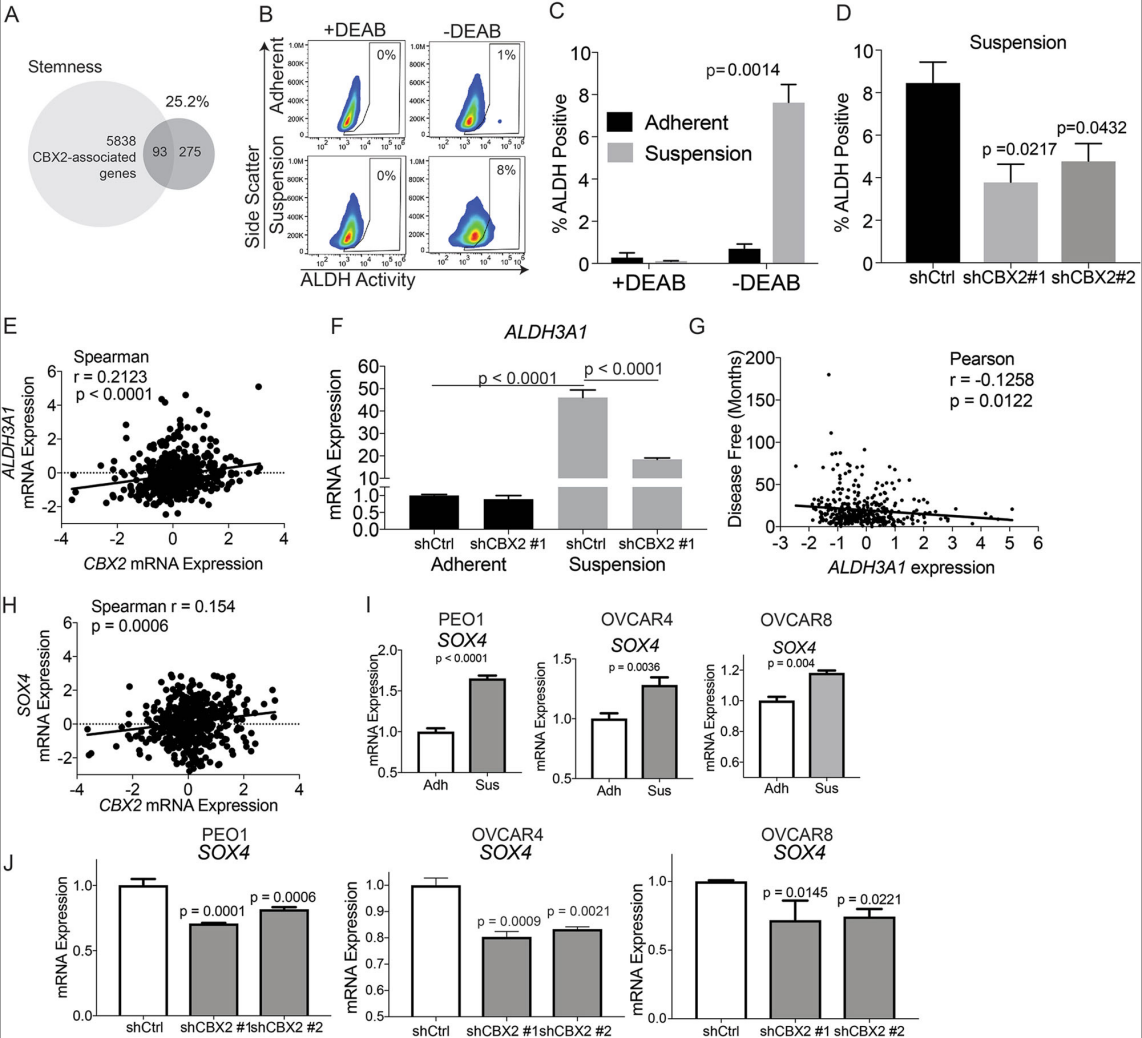
Inhibition of CBX2 decreases stemness. **a** CBX2-associated genes were cross-referenced with a gene set for stemness. Percentage indicates overlap of stemness gene set with CBX2-associated genes. **b** OVCAR4 cells grown in adherent and suspended settings for 7 days. Aldefluor assay and flow cytometry were utilized to determine the percentage of cells that were positive for aldehyde dehydrogenase (ALDH), a marker of stemness. Diethylaminobenzaldehyde (DEAB), a potent ALDH inhibitor, prevented the increase in ALDH activity and served as negative control (left). **c** As above, OVCAR4 cells grown in adherent and suspended settings for 7 days. Bar graph compares the percentage of cells ALDH positive control (+DEAB) to cells without DEAB (experimental) in adherent and suspended settings. Statistical test = two-sided *t*-test. *F* Test *p* = 0.1136. **d** OVCAR4 cells with shControl, shCBX2#1 and #2 cultured in suspension over 7 days. Aldefluor assay and flow cytometry again utilized to determine the percentage of cells ALDH positive. Statistical test = two-sided *t*-test. *F*-test *p* = 0.83 **e** Utilizing the TCGA (HGSOC, Nature, 2011, *n* = 489) dataset, a scatter plot *CBX2* expression was correlated to *ALDH3A1* expression. Spearman correlation *r* = 0.2123 and *p*-value < 0.0001. **f** RT-qPCR of *ALDH3A1* in OVCAR4 cells cultured in adherent and suspension conditions with CBX2 knockdown (shCBX2 #1). Statistical test = ANOVA. **g** Scatter plot of *ALDH3A1* expression (*x*-axis, *Z*-score) compared to disease-free survival (*y*-axis, months). Pearson’s correlation *r* = −0.1258 and *p*-value = 0.0122. **h** Utilizing the TCGA (HGSOC, Nature, 2011, *n* = 489) dataset, a scatter plot *CBX2* expression was correlated to *SOX4* expression. Spearman correlation *r* = 0.154 and *p*-value = 0.0006. **i** PEO1, OVCAR4, and OVCAR8 cells grown in adherent (Adh) or suspension (Sus) settings for 7 days. RNA was extracted, and used for RT-qPCR against *SOX4*. Statistical test = two-sided *t*-test. Experiment performed in technical triplicates and biological duplicate. **j** shControl and shCBX2 #1 and #2 PEO1, OVCAR4, and OVCAR8 were cultured in suspension conditions, RNA was extracted and used for RT-qPCR against *SOX4*. Experiment performed in technical triplicates and biological duplicate. Statistical test = ANOVA. Error bars = S.E.M.

Several reports have demonstrated cells that survive in anchorage-independent conditions possess stem-like characteristics. We hypothesize that the polycomb repressor complex could be involved by inhibiting cellular differentiation and thus maintaining stemness^9,10^. We examined the CBX2-associated genes from TCGA against a published stemness gene set (Supplementary Table 4). We observed 25.2% of stemness-related genes overlapped with CBX2 genes. We next evaluated stemness by measuring aldehyde dehydrogenase (ALDH) activity both in the setting of suspension growth and CBX2 knockdown. We first determined whether placing OVCAR4 and OVCAR8 cells in suspension increased ALDH activity. We observed that in OVCAR4 cells ALDH activity was significantly increased in cells grown in suspension for 7 days (Fig. 6b, c). In contrast, we did not observe an increase in ALDH activity in OVCAR8 cells grown in suspension for 7 days (Supplementary Figure 4A). Next, we evaluated whether CBX2 knockdown impacted suspension-induced ALDH activity, we found that culturing OVCAR4 and OVCAR8 shCBX2 cells in suspension significantly inhibited ALDH activity compared to shControl cells (Fig. 6d and Supplementary Figure 4B-C). These results highlight that the loss of CBX2 regulates ALDH activity in OVCAR4 and OVCAR8 cell lines. The polycomb repressor complex 2 regulates *ALDH1A1* expression^34^, so we sought to determine whether this decrease in ALDH activity correlated with the expression of a specific ALDH gene. We examined the expression of *ALHD1A1, ALDH2, ALDH3A1, ALDH3B1*, and *ALDH6A1* genes in CBX2 knockdown cells (Supplementary Figure 4D-H). *ALDH1A1* was not significantly changed in shCBX2 cells. However, in OVCAR4 and OVCAR8 cells, shCBX2 knockdown cells *ALDH*3*A1* expression was significantly decreased (Supplementary Figure 4H-I). Similar to *ALDH1A1*, *ALDH3A1* has previously been associated with stemness^35^. Consistently, the examination of HGSOC samples in the TCGA revealed CBX2 expression correlated with ALDH genes expression with the *ALDH3A1* gene having the only significant positive correlation (Supplemental Figure 4J and Supplementary Table 5). Further analysis of TCGA data comparing *CBX2* and *ALDH3A1* expression found a significant positive correlation; suggesting CBX2 promotes increased *ALDH3A1* expression (Fig. 6e). In suspension, *ALDH3A1* was significantly upregulated compared to adherent cells and knocking down CBX2 significantly abrogated *ALDH3A1* expression (Fig. 6f and Supplementary Figure 4H-I). Furthermore, ALDH3A1 expression significantly correlated with disease-free survival (Fig. 5g, Pearson’s *r* = −0.1258, *p* = 0.0122). Referring to the TCGA data comparing CBX2 expression we also identified a significant correlation to a potent stem cell-associated transcription factor, SOX4 (Fig. 6h), [reviewed in ref. ^36^]. In adherent vs. suspension settings SOX4 was significantly upregulated in OVCAR4, OVCAR8, and PEO1 cells grown in suspension (Fig. 6i). Consequentially, CBX2 knockdown promoted a significant decrease in *SOX4* expression (Fig. 6j). Taken together, these data strongly suggest that HGSOC cells grown in suspension are more stem-like and CBX2 could be promoting stemness through *ALDH3A1* and *SOX4* regulation.

## Discussion

Several publicly available datasets demonstrate that CBX2 is upregulated in HGSOC, high CBX2 expression portends poorer survival, and increased CBX2 expression correlates with platinum resistance. We confirmed that CBX2 is overexpressed in HGSOC primary tumors, as well as in cell lines that have escaped anoikis in suspension culture. We demonstrated that the loss of CBX2 is associated with decreased proliferation of HGSOC cells in multiple culture conditions, an increase in chemotherapy sensitivity, and a reduction in stem-like cells. Lastly, utilizing a HGSOC tissue microarray of advanced stage primary patient tumors we found CBX2 protein expression was expressed in a majority of tumors.

CBX2 was found to be highly expressed in primary HGSOC tumors from seven patients. Clinically, three of these patients had more extensive peritoneal disease (Supplementary Table 2) suggesting that CBX2 could serve as a predictive marker of advanced disease, reinforcing the potential clinical significance of CBX2. Moreover, examination of five patients with matched primary tumor, ascites-associated tumor cells, and distant metastasis revealed three of the five patients had an increase in *CBX2* expression in distant metastasis/ascites-associated tumor cells compared to primary tumors. This highlights that CBX2 is potentially important in driving HGSOC progression, however there are indeed other contributing factors. Notably we observed that high expression of *CBX2* correlated to a loss of an active tumor suppressor, FOXO3. This suggests that the downregulation of FOXO3 independent of CBX2 could drive tumor progression. As we have attempted to elucidate the mechanism behind CBX2-dependent activity we see common themes of increased proliferation and stem-like differentiation, which appeared to lead to a more aggressive and chemoresistant phenotype. The loss of CBX2 led to the loss of stemness measured through ALDH activity, *ALDH3A1* expression, and *SOX4* expression suggesting that reduced stemness is a major driver of these phenotypes. In addition, we linked the anoikis-induced autophagy, EMT, and apoptosis response to CBX2 expression. Further investigations will functionally evaluate the relationship between CBX2 and these key survival processes.

CBX2 is a subunit of the polycomb repressor complex (PRC1), which has been shown to play a role in ovarian cancer^13,37^. The enzymatic subunit or “writer” of PRC1, BMI-1, is considered to play a role in malignant transformation of multiple cancers, including ovarian cancer^38,39^. In ovarian cancer, BMI-1 has been demonstrated to be associated with stem-ness and tumor initiation and serves as an independent predictor of poor outcome^9,16,40^. Moreover, silencing of BMI-1 can lead to improved sensitivity to chemotherapy^40,41^. This understanding of BMI-1 directly correlates and aligns with our CBX2 findings. Taken together with observations in other types of cancer, it seems likely that the PRC1 and specifically, CBX2, are novel therapeutic targets not only for HGSOC, but potentially for breast and prostate cancers.

CBX2 is considered to be an epigenetic “reader”. The existing literature suggests that targeting epigenetic “readers” is an effective strategy for targeted therapy. Bromodomain (acetyl-histone “reader”) inhibitors have the potential to suppress ALDH activity in ovarian cancer, providing evidence that targeting of an epigenetic reader may be able to alter the stem-like phenotype of a cell^42^. One key example is JQ-1, a potent and selective inhibitor of the bromodomain and extra-terminal domain (BET) family of proteins, including BRD2, BRD3, and BRD4^43^. Preliminary clinical data suggests that BET inhibitors may have therapeutic potential in human cancers^43–46^. Furthermore, a Phase I clinical trial of an oral BET inhibitor demonstrated that this small molecule inhibitor was well tolerated in vivo, a critical development which paves the way for future targeting of epigenetic readers^46^. This highlights that a “reader” or chromobox domain inhibitor could prove to be more effective with fewer adverse effects compared to inhibitors of epigenetic “writer” enzymes.

In this report, we describe the potential role of CBX2 in promoting HGSOC disease progression. Mechanistically, CBX2 protects HGSOC against apoptosis and promotes a more stem-like phenotype. CBX2 is an epigenetic reader and is therefore targetable with a small molecule inhibitor. This work expands our understanding of the progression of HGSOC and identifies a novel therapeutic target.

## Materials and methods

### Cell culture

OVCAR4, PEO1, and OVCAR8 human high grade serous ovarian cancer cell lines were authenticated using small tandem repeat (STR) analysis (The University of Arizona Genetics Core) and routinely tested for mycoplasma with MycoLookOut (Sigma, St. Louis, MO). OVCAR8 and OVCAR4 cells were obtained from the Gynecologic Tumor and Fluid Bank (University of Colorado, Aurora, CO). PEO1 purchased from American Type Culture Collection. Cells were cultured in RPMI-1640 medium supplemented with 1% penicillin–streptomycin and 10% fetal bovine serum. The cell lines were maintained in 5% CO_2_ at 37 °C.

### Bioinformatic database analysis

Gene Expression Omnibus (GEO), hosted by the National Center for Biotechnology Information (NCBI), was queried for relevant databases. GSE18521, GSE10971, GSE1926, and GSE73064 were examined for relative CBX2 expression. The Cancer Genome Atlas (TCGA) Ovarian Serous Cystadenocarcinoma (Nature 2011^46^) database was accessed via the cBIOPortal (http://www.cbioportal.org/) to evaluate the role of CBX2 in HGSOC disease-free and overall survival. Note: patient numbers for overall survival (*n* = 485) and disease-free survival (*n* = 396) differ due to the availability of data with TCGA. The database was queried for HGSOC identifying a total of 557 tumors with mRNA expression data and CBX2 upregulation was defined as CBX2 mRNA expression >1.5 standard deviation. RPPA data was assessed from the HGSOC Provisional dataset via the cBIOPortal.

### Adherent and suspension environments

The adherent environment was created using standard tissue culture dishes. For suspension, tissue culture dishes were covered with 6 mg/ml poly-2-hydroxyethyl methacrylate (Poly-HEMA, Sigma) in 95% ethanol. The plates were incubated under sterile conditions to allow ethanol evaporation, followed by 30 min of ultraviolet light for sterilization. OVCAR4, PEO1, and OVCAR8 cells were cultured in each of these environments for 7 days.

### Cisplatin dose–response

PEO1 and OVCAR4 cells were plated in 96-well plates in both adherent (4000 cells per well) and suspension (6000 cells per well) environments, as described above. These cells were treated over 24 h with increasing concentrations of Cisplatin (0.5–100 µM). 1640 RPMI media and media with 0.9% NaCl were used for control and vehicle control, respectively. Cell viability was assessed using the 3-(4,5-dimethylthiazol-2-yl)-5-(3-carboxymethoxyphenyl)-2-(4-sulfophenyl)-2*H*-tetrazolium (MTT) assay (Promega, Madison, WI). Means of at least five wells are reported and experiments were independently repeated in triplicate. Representative dose–response curves are shown.

### Quantitative reverse transcription polymerase chain reaction (RT-qPCR)

RNA was isolated using RNAeasy Plus Mini Kit (Qiagen, Hilden, Germany) according to the manufacturer's protocol. NanoDrop spectrophotometry was performed to confirm the concentration of extracted RNA. RT-qPCR was performed using the Luna Universal One-step RT-qPCR kit (New England BioLabs, Ipswich, MA) on a BioRad CFX96 or Applied Biosystems QuantStudio 6 Flex thermocycler using primers for specific target transcripts; 18s rRNA was examined as a housekeeping gene (Supplementary Table 6).

### shRNA knockdown

CBX2-specific shRNA were obtained from the University of Colorado Functional Genomics Facility (CBX2 #1: TRCN 0000020327 and CBX2 #2: TRCN 0000020328). An empty pLKO.1-puro was utilized as shControl (shCtrl). Plasmid isolation was performed using Plasmid Midi-Prep Kit (Qiagen). Twenty-four hours after seeding, cells were transfected with a total of 12 µg of DNA, including lentiviral packaging plasmids and the shRNA, in addition to 36 µg of polyethyenimine (PEI), for a 1:3 ratio of DNA to PEI. Cells were incubated overnight and transitioned to Dulbecco’s Modified Eagle Media (DMEM) the following morning. Forty-eight hours after medium change, lentivirus was harvested. PEO1, OVCAR4, and OVCAR8 cells were seeded into six-well plates. When cells reached 80% confluence, they were transduced with lentivirus encoding CBX2-specific shRNAs or an shRNA control. A control well was maintained without virus to confirm puromycin selection. A 48-h puromycin selection was performed immediately following transduction. After medium change, cells were allowed to recover and then subjected to functional assays.

### Immunoblot

Cell lysis was performed using radioimmunoprecipitation assay (RIPA) buffer (150 mM sodium chloride, Triton X-100, 0.5% sodium deoxycholate, 0.1% SDS [sodium dodecyl sulfate], 50 mM Tris, pH 8.0) supplemented with complete EDTA-free protease inhibitor cocktail (Roche), as well as NaF and NaV. Protein was quantified using bicinchoninic acid (BCA) protein assay (Thermo Fisher Scientific, Waltham, MA) and spectrophotometry. An 8% SDS polyacrylamide gel resolving gel was created with a 4% stacking gel. Twenty to thirty micrograms of total protein were loaded per well. Proteins were transferred to PVDF membrane using a Bio-Rad TransBlot Turbo. Western Blot analysis was performed using a rabbit primary antibody specific to CBX2 (Thermo Fisher Scientific, Cat # PA5-30996, 1:1000) and a mouse primary antibody against actin (Abcam, Cat # ab6276). CBX2 previously validated in Clermont Paper^13^. Primary antibody incubation was performed overnight at 4 °C. Secondary goat anti-rabbit green (LI-COR Biosciences, Lincoln, NE, Cat # 926-32211) and goat anti-mouse red (LI-COR, Cat # 926-68070) antibodies were applied the following morning for 1 h at room temperature. Bands were visualized using the LI-COR Odyssey Imaging System.

### Proliferation assays

For the Gaussia luciferase (gLuc) assay, the BioLux Gaussia Luciferase Assay kit (New England BioLabs) was utilized. OVCAR4 and PEO1 cells were grown in a 96-well plate, starting with 2000 cells per well. Media were collected every 24 h and stored in at −20°. For the assay and luminometer readings, the media were thawed and placed in a new 96-well plate. The assay was performed following the manufacturer’s protocol and the relative light units were obtained using luminometry (GloMax) and charted with Prism software. Colony formation assays were performed in parallel using crystal violet staining. Briefly, cells were fixed (10% methanol/10% acetic acid/PBS), stained with crystal violet (0.4%) and washed with de-ionized water. Crystal violet was dissolved and absorbance was measured using a spectrophotometer (SpectraMaxM2e, Molecular Devices, San Jose, CA) at 590 nm and SoftMaxPro software. For the spheroid assay, 4000 cells were plated from a single cell suspension onto growth factor reduced Matrigel (Corning, Corning, NY) and allowed to incubate for 12 days. Microscopic images were obtained and the diameter of each spheroid was measured in ImageJ (NIH). At least 50 spheroids were measured for each cell type and the diameters were averaged and graphed using Prism software.

### Aldefluor assays

Stemness was evaluated using the Aldefluor Kit (Cat # 01700, Stemcell Technologies, Vancouver, Canada). OVCAR4 and OVCAR8 cells grown in adherent and suspension settings for 7 days were collected and prepared following manufacturer’s protocol. An exception to the manufacturer's protocol was the reduction of Aldefluor reagent by 50%, using 2.5 µl per 1 ml of cell suspension. Flow cytometry was performed on a Gallios 561 Cytometer (BD Biosciences) with analysis at 488 nm.

### Gynecologic Tissue and Fluid Bank (GTFB)

The University of Colorado has an Institutional Review Board approved protocol (COMIRB #07-935) in place to collect tissue from gynecologic patients with both malignant and benign disease processes. All participants are counseled regarding the potential uses of their tissue and sign a consent form approved by the Colorado Multiple Institutional Review Board. The tissues are processed, aliquoted, and stored at −80 °C.

### Immunohistochemistry

Patient-derived xenograft tissue samples of HGSOC were fixed in formalin and embedded in paraffin. The University of Colorado Cancer Center Histology Core performed serial sectioning of the tissue at 5 micron thickness. For histopathologic examination, sections were de-paraffinized using xylene and hydrated in graded alcohol solutions. Antigen retrieval was performed using citrate buffer (pH 6.0) and boiling in pressurized steamer to 110 °C for 30 min. Endogenous peroxidase activity was quenched with 3% hydrogen peroxide in methanol for 20 min, followed by washing in TBS. A hydrophobic barrier was drawn around each section and tissues were blocked in 1% BSA in TBS for 30 min. TMA slides were single stained. Rabbit anti-CBX2 (Thermo Scientific, Cat # PA5-30996) was diluted to 1:50 in 1% BSA in TBS, applied to all sections, and incubated overnight at 4 °C. Rabbit anti-PAX8 (Proteintech, Cat # 10336-1-AP) was diluted to 1:200 in 1% BSA in TBS, applied to all sections, and incubated overnight at 4 °C. An isotype control (Rabbit IgG) was incubated in parallel. The secondary antibody, anti-rabbit Dako Envision+ System HRP Labeled Polymer (Dako Ref#K4003) was applied to the sections and allowed to incubate for 60 min at room temperature. Slides were subsequently washed with TBS and developed under the microscope using Liquid 3,3′-diaminobenzidine tetrahydrochloride (DAB) + Substrate Chromagen System (Agilent, Santa Clara, CA; Ref#K3468). Slides were counterstained with hematoxylin.

### Tissue microarray

A previously constructed TMA comprised of matched primary, lymph node, and peritoneal metastases samples in duplicate from 24 patients with high grade serous carcinoma treated at the University of Colorado (COMIRB #14-0427), was stained with the CBX2 and PAX8 antibodies. With the aid of the University of Colorado Histology Core, the stained slides were scanned using Aperio imaging technology and annotated to highlight tumor based on PAX8 staining using ImageScope software. The TMA was then analyzed and scored by the University of Colorado Histology Core. Subsequently, two board-certified pathologists (M.D.P. and A.A.B.) manually reviewed and scored the CBX2 stain. Each sample was given a score for intensity (0, 1+, 2+, 3+) and percentage of cells staining (continuous variable). Only distinct nuclear staining of tumor cells was considered positive. From this data, *H*-scores were generated.

### Apoptosis assays

Following 72 h of growth in adherent and suspended states, OVCAR4 and PEO1 cells were harvested and washed in PBS. Alexa 488 Conjugated AnnexinV and Propidium Iodide (PI) (Thermo Fisher Scientific) staining were performed following manufacturer’s protocol.

### Statistical analysis

Prism GraphPad Prism software (v7) was utilized to generate graphs. Statistical tests include unpaired two-sided *t*-tests (comparing two groups), Log-rank (survival) or ANOVA (comparing greater than two groups) unless noted. A significance threshold was set at *p* < 0.05, which was used for sample size determination. All experiments were performed in technical triplicates and biological triplicates unless noted. FloJo software (BD Biosciences, San Jose, CA) was used for analyzing flow cytometry data.

## Electronic supplementary material

Supplementary Figure Legends

Supplementary Figure 1

Supplementary Figure 2

Supplementary Figure 3

Supplementary Figure 4

Supplementary Table 1

Supplementary Table 2

Supplementary Table 3

Supplementary Table 4

Supplementary Table 5

Supplementary Table 6
