# Supplementary material for: CBX2 identified as driver of anoikis escape and dissemination in high grade serous ovarian cancer
Source: Oncogenesis. 2018 Nov 26;7(11):92. doi: 10.1038/s41389-018-0103-1 (PMC6255906; doi:10.1038/s41389-018-0103-1)
Supplement: Supplementary file 1 — Supplementary Figure Legends [file 41389_2018_103_MOESM1_ESM.docx]

Supplementary Figure Legends

**Supplementary Figure 1: Suspension growth and CBX2 modulation in multiple cell lines.** **A:** OVCAR8 grown in adherent and suspension settings (described in Figure 2A) over 7 days. Protein utilized for immunoblot against CBX2. **B:** RT-qPCR for *CBX2* in PEO1 cells transduced with small hairpin RNA control (shCtrl) or specific for *CBX* (shCBX2 #1 and #2). Internal control = *18s*. Statistical test = ANOVA. **C:** Protein derived from PEO1 shControl (shCtrl), shCBX2#1, and shCBX2#2 transduced cells utilized for immunoblot against CBX2 (actin=loading control). **D**: Same as B, but examined CBX2 mRNA expression in OVCAR8 cells. Internal control = *18s*. Statistical test = ANOVA. **E:** Experimental confirmation of Gaussia luciferase (gLuc) assay. OVSAHO cells were transduced with gLuc virus and selected with puromycin. Known number of cells grown over 24 hours, media collected, gLuc assay performed (left). Equivalent number of cells seeded across 24 well plate, media collected and assayed over hours (middle) and days (right). Relative luminescence units by gLuc increases with number of cells. Linear regression r^2^ indicated. **F:** Proliferation assay of PEO1 cells with CBX2 knockdown and scramble control, grown in adherent setting (tissue culture plastic), measured by gLuc activity every 24hr for 96hr. Calculated as proliferation rate: gLuc relative intensity per hour. Statistical test = ANOVA. **G:** Same as E, PEO1 cells grow in suspension setting with utilization of polyHEMA coated plates (Figure 2A). Proliferation rate calculated. Statistical test = ANOVA. **G:** PEO1 cell lines (shControl [shCtrl], shCBX2 #1 and #2) grown in 3D using Matrigel over 12 days, forcing spheroid growth. Spheroids measured across horizontal diameter. Mean calculated from all measurements. Representative images of spheroids below X axis. Scale Bar = 100µm. Statistical test = ANOVA. **I:** ShCtrl, shCBX2 #1 and #2 PEO1 cells grown in adherent or suspension were utilized for an AnnexinV/PI assay. Percentage positive AnnexinV/PI graphed. Statistical test = ANOVA. **J:** Same as I, but examined OVCAR8 cells. Error bars=S.E.M.

**Supplementary Figure 2: CBX2 antibody validation for immunohistochemistry.** Serial sections of a HGSOC tumors were utilized for IHC against a negative control, a matched isotype (Rb IgG), and CBX2 (brown). As expected a majority of the CBX2 localized to the nucleus (white arrows). Scale bars = 100 µm.

**Supplementary Figure 3: CBX2 knockdown sensitizes OVCAR8 and PEO1 cells to cisplatin. A:** shControl (shCtrl), shCBX2 #1 and #2 OVCAR8 cells grown in adherent were dosed with cisplatin for 24 hours. Treated cells were utilized for a MTT assay to assess cell viability. **B**: Same as A, but adherent PEO1 cells were dosed with cisplatin for 48 hours. **C:** Same as A, but OVCAR8 cells in suspension were dosed with cisplatin for 24 hours. **D:** Same as A, but PEO1 cells in suspension were dosed with cisplatin for 48 hours. IC_50_ values were calculated with Prism and are indicated.

**Supplementary Figure 4: CBX2 knockdown leads to loss of ALDH3A1 expression. A:** OVCAR8 cells were grown in adherent and suspension conditions for 7 days and utilized for ALDHfluor assay. Percentage of ALDH positive cell graphed. Statistical test = t-test. **B**: shControl (shCtrl), shCBX2 #1 and #2 OVCAR8 cells were grown in adherent condition for 7 days and utilized for ALDHfluor assay. Percentage of ALDH positive cell graphed. Statistical test = ANOVA. **C**: Same as B, but examined ALDH positive OVCAR8 cells grown in suspension for 7 days. Statistical test = ANOVA. **D:** RT-qPCR for *ALDH1A1* in OVCAR4 cells transduced with shControl (shCtrl) or shCBX2 #1 and #2. RNA was collected from adherent cells and used for RT-qPCR against CBX2. **E**: Same as D, but RT-qPCR for *ALDH6A1*. **F**: Same as D, but RT-qPCR for *ALDH2*. **G**: Same as D, but RT-qPCR for *ALDH3B1*. **H**: Same as D, but RT-qPCR for ALDH3A1. Statistical test = ANOVA. **I**: Same as D, but examined OVCAR cells and RT-qPCR for *ALDH3A1*. Statistical test = ANOVA. Note: All RT-qPCR *18s* was utilized as an internal control. **J**: TCGA (Nature, 2011, n=489) analysis of HGSOC examining correlation between *CBX2* expression and indicated *ALDHs*. Pearson and Spearman correlations (r values) shown in heatmap. Red = positive correlations and Green = negative correlations.
