# Supplementary figures and images for: CBX2 identified as driver of anoikis escape and dissemination in high grade serous ovarian cancer
Source: Oncogenesis. 2018 Nov 26;7(11):92. doi: 10.1038/s41389-018-0103-1 (PMC6255906; doi:10.1038/s41389-018-0103-1)

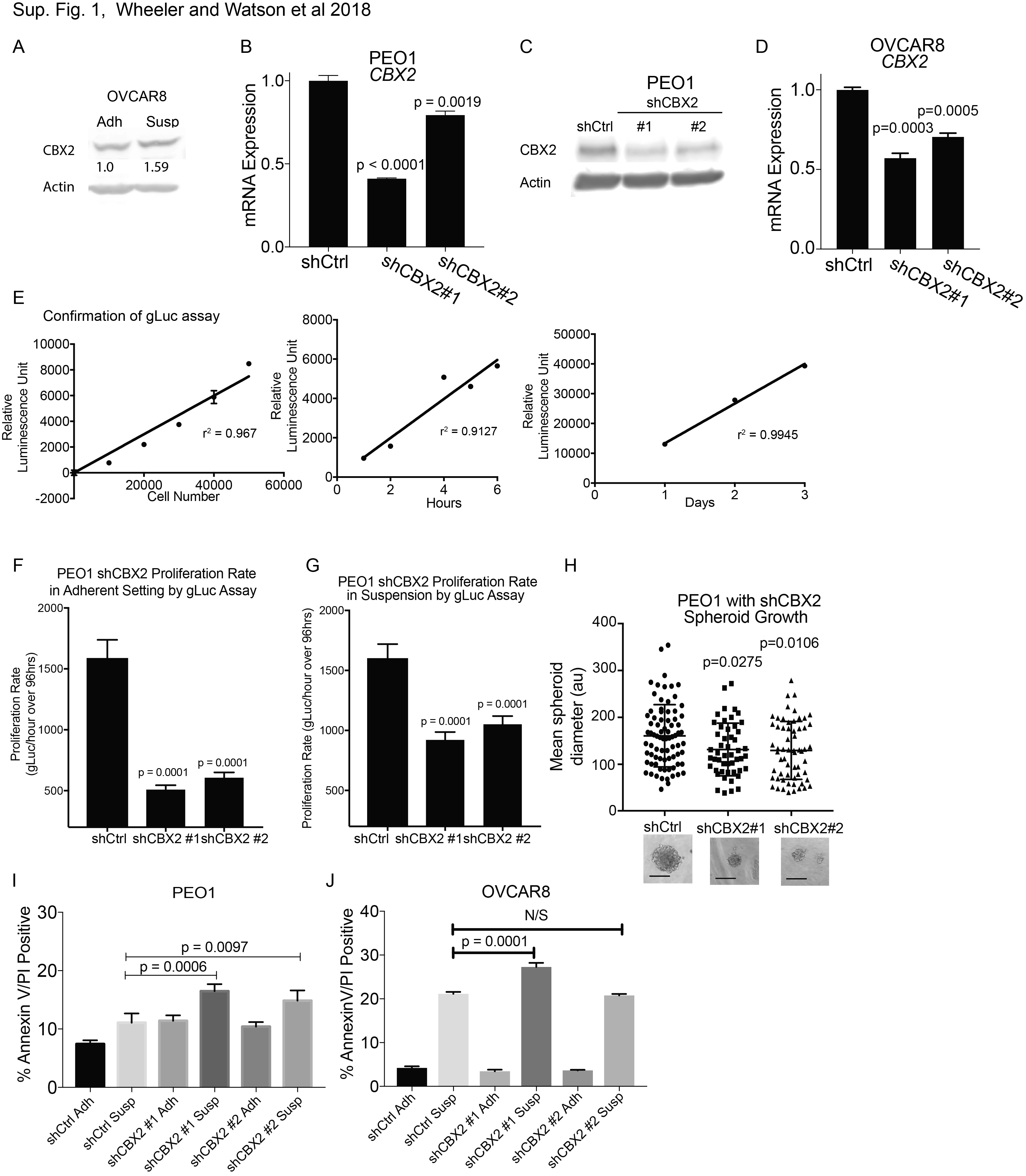

Supplement: Supplementary file 2 — Supplementary Figure 1 [file 41389_2018_103_MOESM2_ESM.jpg]

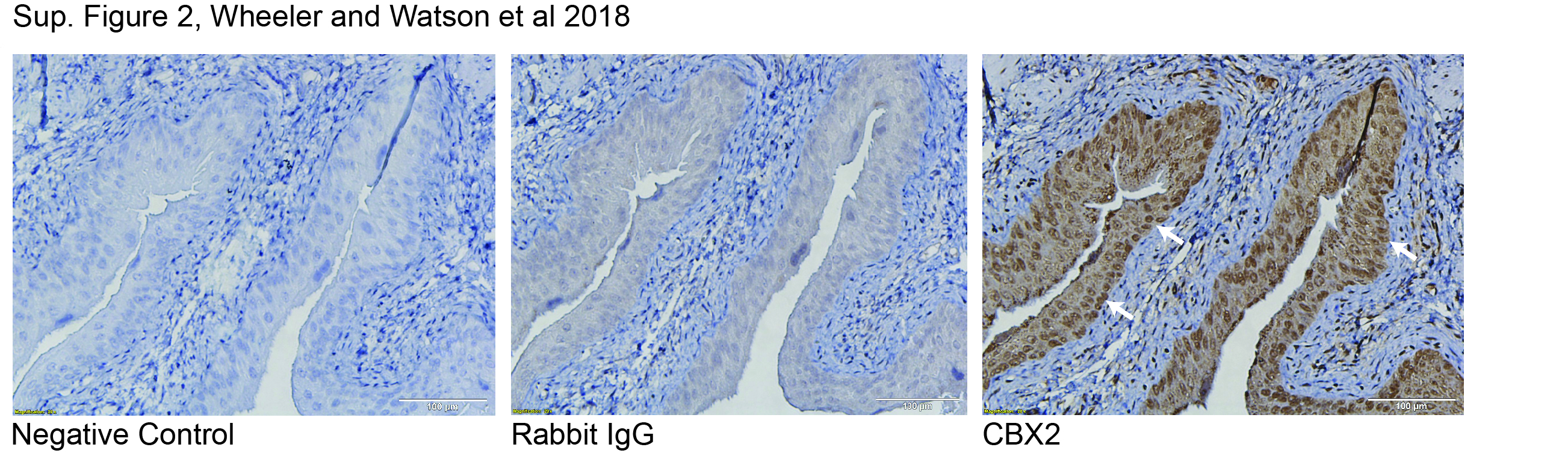

Supplement: Supplementary file 3 — Supplementary Figure 2 [file 41389_2018_103_MOESM3_ESM.jpg]

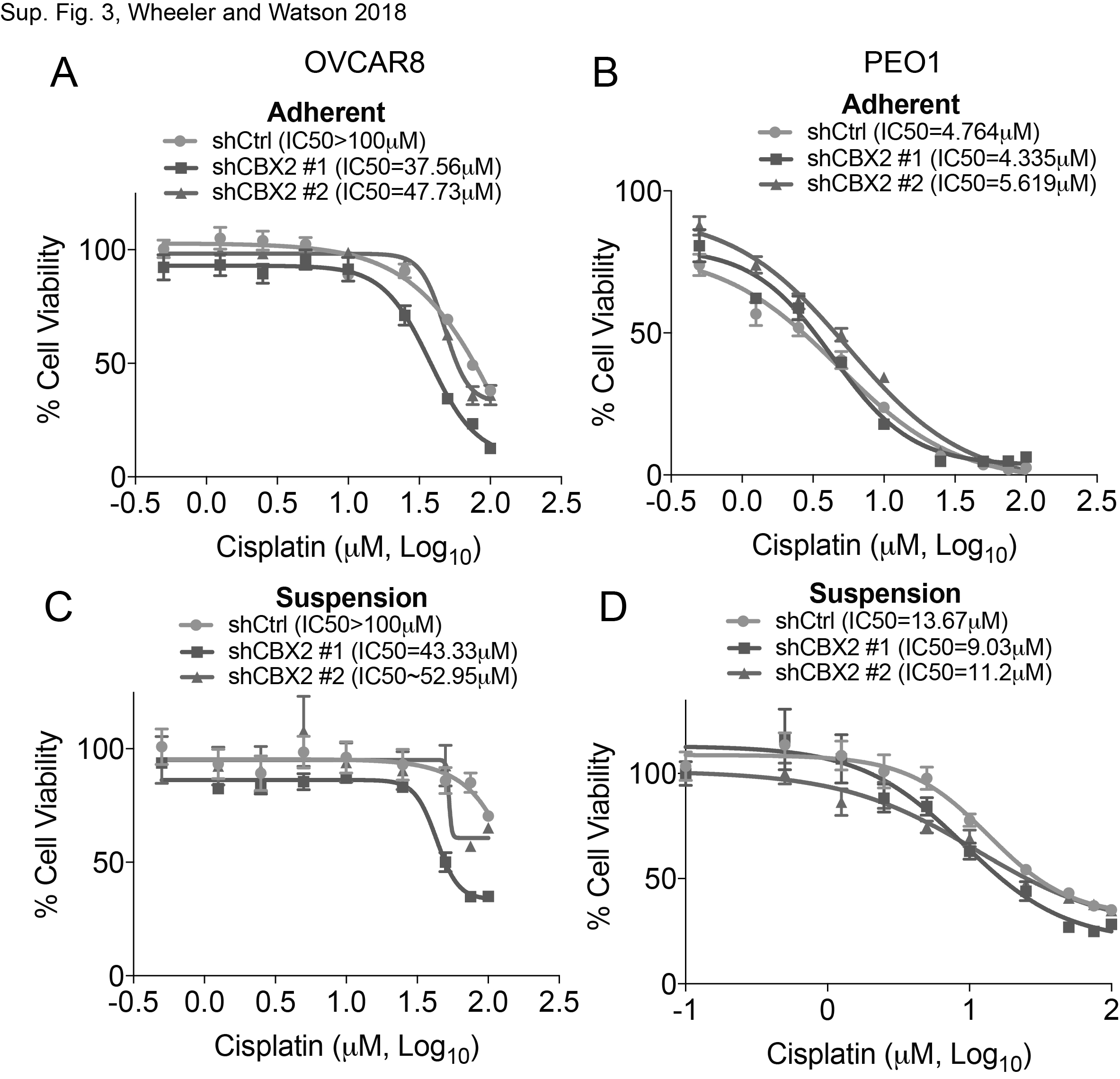

Supplement: Supplementary file 4 — Supplementary Figure 3 [file 41389_2018_103_MOESM4_ESM.jpg]

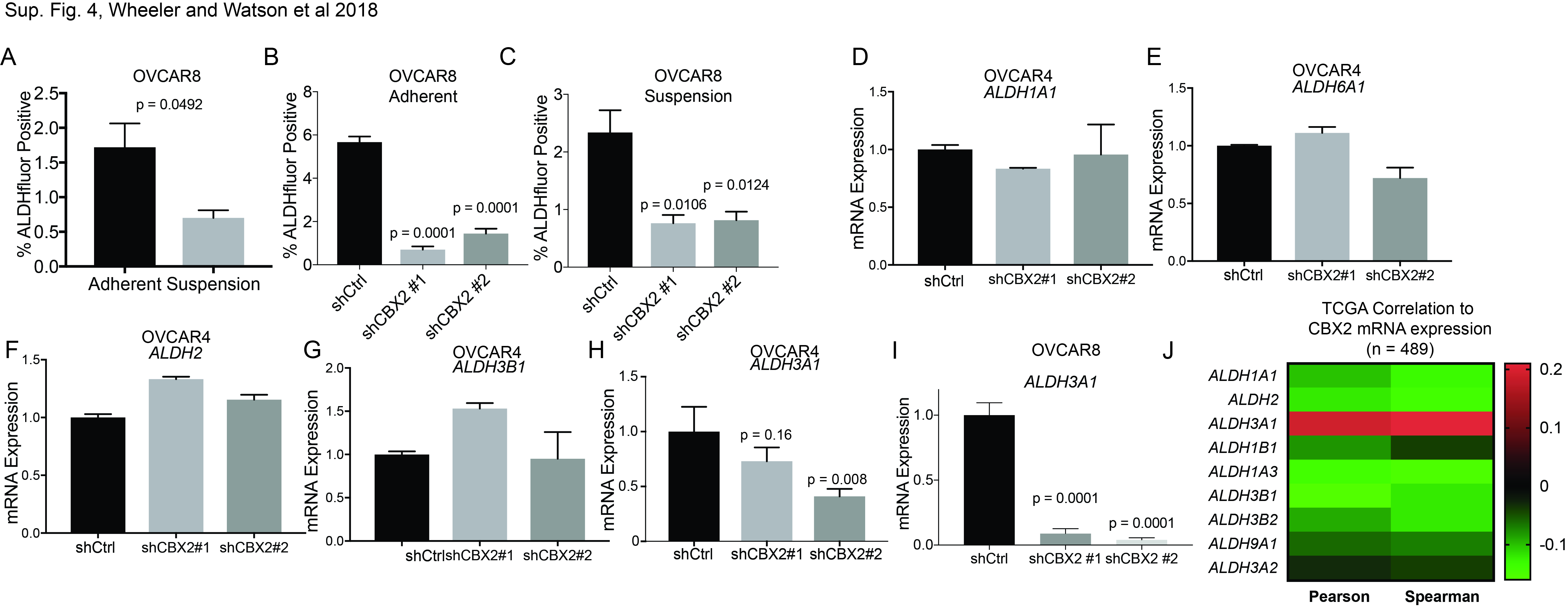

Supplement: Supplementary file 5 — Supplementary Figure 4 [file 41389_2018_103_MOESM5_ESM.jpg]
